# Influence of Abscisic Acid-Biosynthesis Inhibitor Fluridone on the Feeding Behavior and Fecundity of *Nilaparvata lugens*

**DOI:** 10.3390/insects10020057

**Published:** 2019-02-19

**Authors:** Xu Ding, Xi Huang, Litong Sun, Jincai Wu, Jinglan Liu

**Affiliations:** College of Horticulture and Plant Protection, Yangzhou University, Yangzhou 225009, China; dingxu115413@163.com (X.D.); 13218331972@163.com (X.H.); a18851449978@163.com (L.S.); jincaiwu246@163.com (J.W.)

**Keywords:** fluridone, rice, *Nilaparvata lugens*, EPG, fecundity

## Abstract

Fluridone (FLU) was a pyrrolidone herbicide that was used for selective weeding in wheat, rice, corn and pasture and was also a biosynthesis inhibitor of abscisic acid (ABA), a significant plant hormone. ABA-promoted callose deposition facilitates rice resistance to pests but whether FLU had the opposite influence was unknown. The effects of FLU on the feeding behavior of the brown planthopper (*Nilaparvata lugens* Stål; BPH), after feeding with rice plants treated with FLU, were studied, using an electrical penetration graph (EPG). For susceptible rice cultivar (TN1), the duration for which BPH sucked phloem sap (N4 wave duration) after 15 μmol/L of FLU treatment was longer than that of the control but decreased after 30 and 60 μmol/L FLU treatments. Fecundity of BPH treated with 15 μmol/L FLU had no significant change, while the deposition area of callose was significantly decreased. For moderately-resistant rice cultivar (IR42), no differences in BPH feeding behavior and fecundity were observed but the deposition area of callose declined after treated with 15 μmol/L of FLU. These findings suggested that a low concentration of FLU (15 μmol/L) promoted BPH feeding behavior in TN1 but not in IR42 and the response in IR42 appeared to be more complicated, which provided supplementary evidence that ABA promoted plant resistance to BPH.

## 1. Introduction

Abscisic acid (ABA) often activates plant cells to promote the immune system to enhance resistance to abiotic stresses such as salt, drought, cold and other characteristics. In general, ABA is known as a stress hormone. Studies have indicated that ABA had a strong priming effect on the adaptive responses of rice to salt stress [1]. ABA could increase relative water content, reduce damage by stressors to cell membranes and play an important regulatory role in the response to all kinds of biotic stresses [2,3]. For example, You et al. found that ABA promoted the formation of callose in *Arabidopsis thaliana* after biotic stress [4]. Liu et al. reported that the expression of vitellogenin in the brown planthopper (*Nilaparvata lugens* Stål; BPH) (*Nlvg*) was significantly reduced after exogenous ABA treatments [5]. BPH is a piercing-sucking pest with monophagous characteristics in China and Southeast Asia. Fluridone (FLU) is a pyrrolidone herbicide that is used for selective weeding in wheat, rice, corn and pasture [6]. It is a biosynthesis inhibitor of ABA which is mainly achieved by indirect routes through carotenoid synthesis pathways [7,8,9,10,11]. Further studies showed that rice callus treated with FLU had a significantly reduced tolerance to osmotic, salinity or freezing stress. When rice plants were exposed to exogenous ABA treatments and simultaneously treated with FLU, the decrease in rice vitality was completely eliminated [12]. Furthermore, Kuluev et al. provided evidence that the *NtEXGT* (one of Xyloglucan endotransglucosylases/hydrolases (XTHs) of *Nicotiana tabacum*), regulated by ABA, may be involved in ABA-dependent signaling stress factors and the transcriptional level of the *NtEXGT* decreased under drought stress following FLU treatment [13].

Electrical penetration graphs (EPG) have been used as an effective tool to explore the feeding behaviors of insects. The feeding behavior of BPH has been assessed using the EPG method [14,15]. Callose was deposited between cell membranes and cell walls to form physical barriers of papillary structures, which enhanced plant resistance [16]. Hao et al. discovered that BPH feeding induced callose formation in the vascular bundles of rice plants and callose deposition at the stylet entry point of sieve tubes hindered the transport of nutrients [14]. ABA and exogenous ABA induced the formation of callose [17,18,19,20,21,22,23]. Recently, Liu et al. found that ABA promoted the deposition of callose and that impeded BPH from feeding phloem sap [15]. Ovarian anatomy was an important measure for defining the nature of migratory insects, studying the occurrence regularity of pests and predicting their occurrence [24] and also an important experimental technique for studying insect reproduction [25,26]. However, as an ABA inhibitor, the effect of FLU on plant resistance has not been reported. In this paper, we used EPG to measure BPH feeding behavior and we also studied the fecundity of BPH with oviposition period, BPH eggs, relative expression of *Nlvg* and ovarian anatomy. In addtion, we observed the deposition of callose in rice plants treated with FLU in order to further explore the effect of ABA on rice resistance to BPH.

## 2. Materials and Methods

### 2.1. Rice Cultivars and Insects

Rice cultivars, the Taichung Native one (TN1) and IR42 cultivars (China Rice Research Institute Hangzhou, China) were used as BPH susceptible and moderately resistant lines respectively. BPH was collected from the China Rice Research Institute (Hangzhou, China) and reared in the laboratory in an intelligent artificial climate box at Yangzhou University (26 ± 2 °C, humidity 70–80%, light cycle 16 h/8 h). When the external environment was suitable, BPHs were transferred to the experimental field and used in the experiment after three generations.

### 2.2. Analysis of BPH Feeding Behavior with EPG

The EPG technology was determined using the method of Liu et al. [15]. The waveforms of the EPG included NP, N1, N2, N3, N4 and N5 and they had different biological meanings: The NP wave represented non penetration stage; the N1 wave represented the stylet penetrating into the rice epidermis; the N2 wave represented the stage of salivation and the movement of the stylet; the N3 wave represented the extracellular movement of stylet near the phloem region; the N4 wave represented sap ingestion in the phloem; and the N5 wave represented water ingestion in the xylem. When rice seedlings grew to the 4-leaf stage, they were transplanted into plastic cups. Each plastic cup contained one seedling with consistent growth. The rice was sprayed with 10 mL of FLU (15, 30 and 60 μmol/L, Sigma-Aldrich laborchemikalien GmbH D-30918 seelze) with a Jacto sprayer equipped with a cone nozzle for 4 continuous days in the afternoon (Maquinas Agricolas Jacto S.A., Brazil). BPHs were collected from the experimental field and starved for 1 h; BPH bristles were adhered to a 20-μm diameter and 2–3-cm-long gold wire with a soluble conductive adhesive and then connected to an 8-channel insect-stimulated signal electronic recorder (Model: CR-8 DC-EPGI, purchased from Wuhan Precise Electronic Technology Co., Ltd., Wuhan, China). After clicking on the acquisition button, BPH was placed on the rice stem for feeding. The recording process was carried out for 6 h in a shielding case to observe BPH feeding waveforms. One BPH and one seedling were used as a replicate. Each treatment was replicated nine times.

### 2.3. Effects of FLU on BPH Eggs and Oviposition Period

TN1 and IR42 rice cultivars were sprayed with 10 mL of 15 μmol/L FLU at the tillering stage using the above method. Rice plants sprayed with water were used as the controls. Then, third-instar BPH nymphs were released onto the rice plants treated with FLU until mature. Subsequently, some females were collected to determine female reproduction. Females were paired with males and the number of eggs laid and oviposition periods were recorded (one female and one male as a group, 18 groups per concentration). The number of eggs laid per female was recorded every day for 22 days using a microscope. The other females were used to observe the morphology and ovarian development using a microscope.

### 2.4. Ovarian Anatomy

The ovarian anatomy was determined using the method described by Ge et al. [27]. 1× phosphate buffered saline (PBS) was configured with NaCl (137 mM), KCl (2.68 mM), KH_2_PO_4_ (1.47 mM), Na_2_HPO_4_ (8.10 mM) and the pH was adjusted to 7.0. The mated females raised on TN1 and IR42 rice cultivars after 15 μmol/L FLU treatments were put in 1× PBS and their ovaries were collected with dissecting forceps. At room temperature, the ovaries were put into 1× PBS (including 3.8% formaldehyde) for 20 min, followed by three times washing with 0.2% Triton-X 100 (Sigma-Aldrich Inc., St. Louis, MO, USA) in 1× PBS for 10 min. Finally, the ovaries were observed with an Olympus microscope (Olympus Co., Ltd., Ishikawa, Japan) and photographed with a Fuji FinePix S2 Pro digital camera (Fujifilm Co., Ltd., Tokyo, Japan). The anatomy of the ovaries from twelve females for each treatment were observed using a microscope.

### 2.5. Influences of FLU on the Relative Expression of Nlvg

Three BPH adult females were used as a replicate and three repetitions were set up per treatment. Total RNA was extracted from the BPH females with TaKaRa MiniBEST Universal RNA Extraction Kit (Takara Bio Inc., Kusatsu, Japan). Reverse transcription was performed with the PrimeScriptTM RT reagent Kit (Takara Bio Inc., Kusatsu, Japan) with gDNA Eraser (Perfect Real Time). The cDNA reverse transcription reaction conditions were 37 °C for 15 min, 85 °C for 5 s and 4 °C for 15 min. qRT-PCR was used to determine the changes in mRNA contents. A three-step method was used with SYBR Premix Ex TaqTM II (Tli RNaseH Plus) (Takara Bio Inc., Kusatsu, Japan) reagent. Each sample was subjected to 3 replications. The qRT-PCR amplification procedure was as follows: 50 °C for 5 min, then 95 °C for 5 s, 56 °C for 30 s and 72 °C for 30 s, 40 cycles are carried out in total. Here are the qRT-PCR primers: Vg-F: GTGGCTCGTTCAAGGTTATGG, Vg-R: GCAATCTCTGGGTGCTGTTG; β-F: TGGACTTCGAGCAGGAAATGG, β-R: ACGTCGCACTTCAGATCGAG.

### 2.6. Callose Deposition Area in Rice after Treated with FLU

At the tillering stage, TN1 and IR42 rice cultivars were sprayed with 10 mL of FLU (15 μmol/L) using the above method. After BPH feeding for 1, 2 and 3 days, rice leaf sheaths were collected as a sample and stored in an ultra-low-temperature freezer. The sheaths were cut into 0.3–0.5 cm pieces after being washed and dried. Then, the pieces were placed in 10% glycerin and agitated for approximately 15 min until they sank, followed with fixing and slicing at −50 °C. A brush was used to move the slice to a slide. The slice was allowed to dry slightly at room temperature, soaked in 96% alcohol for 6–10 h. After the alcohol dried, 1 mL of 1/15 mol/L phosphate buffer was added to the slides. After 45 min, 1 mL aniline blue staining solution of 0.1% was added and dyed for 60 min. After the slides were dried, the callose deposition area in the vascular bundles was observed with UV light under an Olympus BX51 fluorescence microscope (Olympus Co., Ltd., Ishikawa, Japan) and photographed with a microscope-specific camera. According to the number of vascular bundles and their site and area, the callose deposition area of each bundle was calculated with 10 replicates for each treatment.

### 2.7. Data Analysis

The statistical significance of the difference between treatments was analyzed using analysis of variance (ANOVA; Systat Inc.). Multiple comparisons were conducted using the *PLSD* test. The data were denoted as means + SE and analyzed using SPSS 11.0 software [28]. Callose deposition area was calculated by Image-Por Plus 6.0 image analysis software (Media Cybernetics Inc., Bethesda, MA, USA). Based on the number of vascular bundles examined, the relative area in each vascular bundle was calculated.

## 3. Results

### 3.1. EPG Analysis of BPH Feeding Behavior

**Figure 1:**
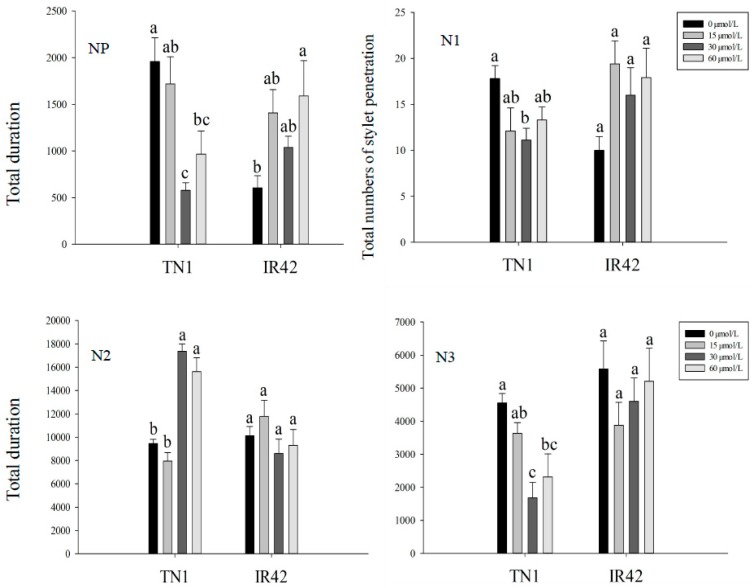

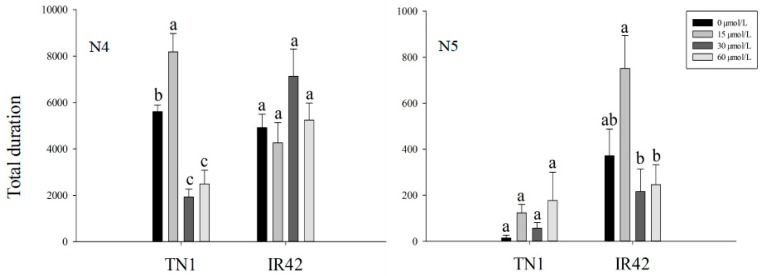
Brown planthopper (BPH) feeding behavior was analyzed after feeding TN1 and IR42 rice cultivars treated with 15, 30 and 60 μmol/L FLU. NP wave showed the total duration of non-penetration stage. N1 wave showed the total number of stylet penetrating into rice epidermis. N2 wave showed the duration of salivation and movement of stylet. N3 wave showed the duration of extracellular movement of stylet near the phloem region. N4 wave showed the duration of ingesting sap in phloem. N5 wave showed the duration of ingesting water in xylem. Different letters in the bars showed the means of the same parameters differed significantly under the same rice cultivar and different FLU treatment at the *p* < 0.05 level.

As shown in Figure 1, the duration of the NP wave (non-probing) on the TN1 rice cultivar treated with 30 and 60 μmol/L FLU was significantly shorter than that of the control. The total number of the stylets penetrating into the rice epidermis on the TN1 rice cultivar treated with 30 μmol/L FLU was significantly less than that of the control, with a decrease of 37.6% (*F* = 2.95, *df* = 3, 35, *p* < 0.05). The duration of the N2 wave (treated with 30 and 60 μmol/L) was significantly longer than that of the control, increasing by 83.5% and 65.2%, (*F* = 34.37, *df* = 3, 35, *p* < 0.05) respectively. The duration of the N3 wave was significantly lower than that of the control, decreasing by 63.1% (30 μmol/L FLU) and 29.2% (60 μmol/L FLU) (*F* = 7.58, *df* = 3, 35, *p* < 0.05). The N4 wave (sap ingestion in phloem) of the TN1 treated with 15 μmol/L FLU was the longest, increasing by 45.7% compared to the control (*F* = 28.46, *df* = 3, 35, *p* < 0.05) but decreased after 30 and 60 μmol/L FLU treatments and decreasing rate was 65.7% and 55.7%, respectively (*F* = 28.46, *df* = 3, 35, *p* < 0.05). No significant difference in the duration of the N5 (water ingestion in xylem) wave among 0, 15, 30 and 60 μmol/L FLU treatments was observed (Figure 1).

The duration of the NP wave in the IR42 rice cultivar treated with 60 μmol/L FLU was significantly longer than that of the control, with an increase of 229.1%. For N1, N2, N3, N4 and N5 waves, after 15, 30 and 60 μmol/L FLU treatments, no significant difference had been found compared with the control. The duration of the N5 wave on the IR42 rice cultivar treated with 15 μmol/L FLU was significantly longer than that of the 30 and 60 μmol/L FLU treatments (Figure 1).

### 3.2. Effects of FLU on the Fecundity of BPH

**Figure 2:**
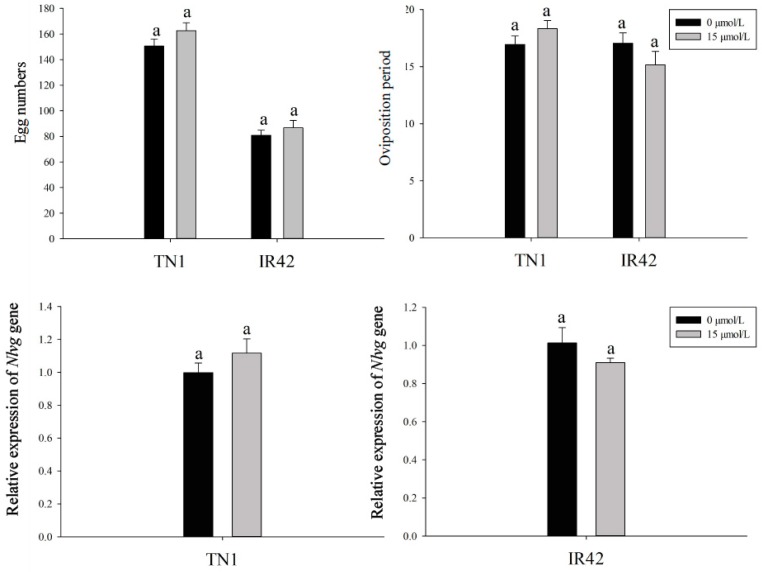
Changes of the fecundity, oviposition period and the expression level of *Nlvg* in female BPH after feeding TN1 and IR42 rice cultivars treated with FLU. Data was presented as means + SE. Means followed in the same rice cultivar by different letters indicated significant difference at the 5% level (*PLSD* test, *p* < 0.05 level). Different letters in the bars showed the means of the same parameters differed significantly under the same rice cultivar at the *p* < 0.05 level.

The number of eggs laid by BPH females after feeding on the rice cultivars treated with 15 μmol/L FLU and the control were 162.7 and 150.6 for the TN1 rice cultivar, 86.7 and 80.8 for the IR42 rice cultivar, respectively (Figure 2) and the oviposition periods were 18.3 and 16.9 for the TN1 rice cultivar, 17.01 and 15.17 for the IR42 rice cultivar, respectively (Figure 2). No significant differences were observed in the fecundity of BPH after feeding on the rice cultivars treated with 15 μmol/L FLU and the control. Similarly, after feeding on the rice cultivars treated by 15 μmol/L FLU, the expression level of *Nlvg* was as presented in Figure 2 and no significant changes were found compared with the control (Figure 2), which was consistent with the amount of eggs laid and the oviposition periods in BPH.

**Figure 3:**
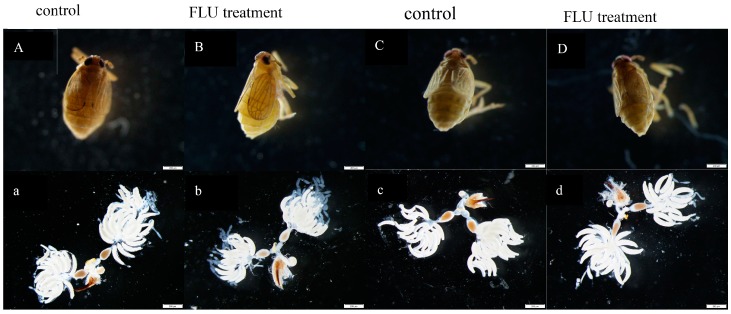
The morphology and ovarian development of BPH after feeding TN1 (**A**,**B** and **a**,**b**) and IR42 (**C**,**D** and **c**,**d**) rice cultivars treated with 15 μmol/L FLU. **A** (**a**), control (0 μmol/L FLU treatment), TN1; **B** (**b**), 15 μmol/L FLU treatment, TN1; **C** (**c**), control (0 μmol/L FLU treatment), IR42; **D** (**d**), 15 μmol/L FLU treatment, IR42. Body size (**A**–**D**) and reproductive tracts (**a**–**d**) were photographed with a Leica DMR from twelve females for each treatment. Bars = 200 μm.

The ovarioles within the ovaries of BPH after feeding with rice cultivars treated with FLU and control females contained two ripe banana-shaped oocytes; there was no significant change in the morphology and ovarian development in BPH after feeding with the rice cultivars treated with FLU compared to the control (Figure 3).

### 3.3. Effect of FLU on Callose Deposition in Rice Cultivars

**Figure 4:**
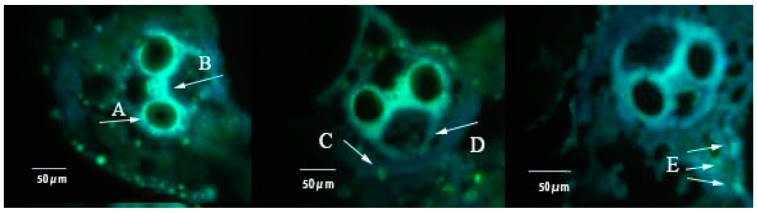
The yellowish green fluorescence light spot showed the callose deposition in rice sheath tissues under a fluorescence upright microscope. (**A**) callose deposition in duct; (**B**) callose deposition in xylem tissue; (**C**) callose deposition in epidermal tissue; (**D**) callose deposition in bundle sheath; (**E**) callose deposition in parenchyma, Bars = 50 μm).

**Figure 5:**
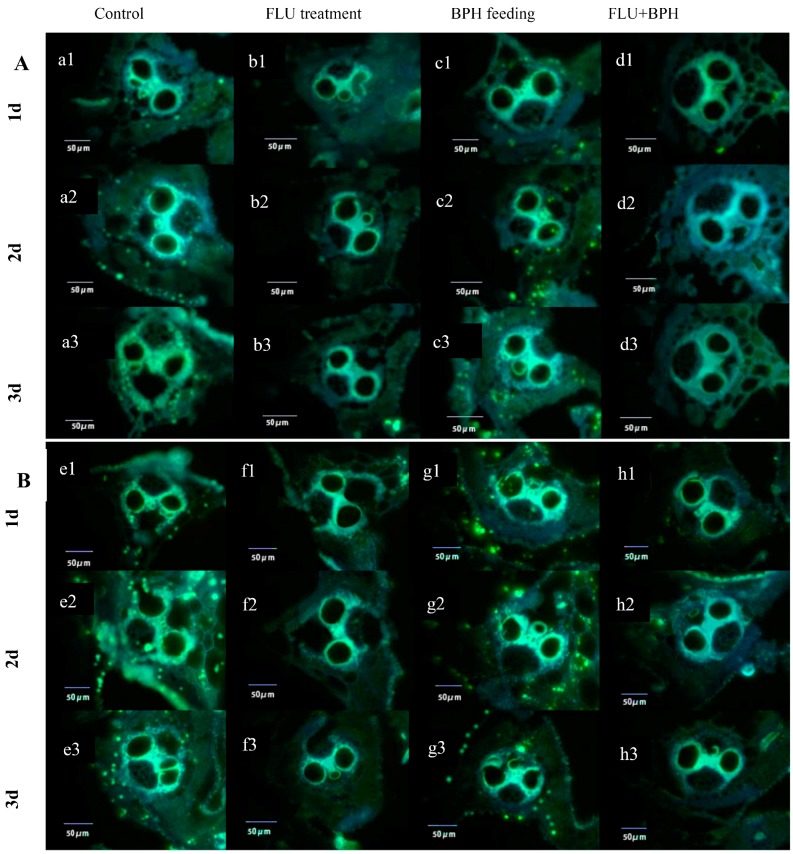
The callose deposition area in TN1 and IR42 rice cultivars after 15 μmol/L FLU treatment and BPH feeding for 1, 2 and 3 days. The yellowish green fluorescence light spot showed the callose deposition in TN1 (**A**) and IR42 (**B**) rice leaf sheath tissues under a fluorescence upright microscope (Bars = 50 μm) after FLU treatment and BPH feeding. a1, a2, a3 and e1, e2, e3 (control, without FLU treatment and no BPH feeding); b1, b2, b3 and f1, f2, f3 (15 μmol/L FLU treatment); c1, c2, c3 and g1, g2, g3 (BPH feeding); d1, d2, d3 and h1, h2, h3 (15 μmol/L FLU treatment and BPH feeding).

**Figure 6:**
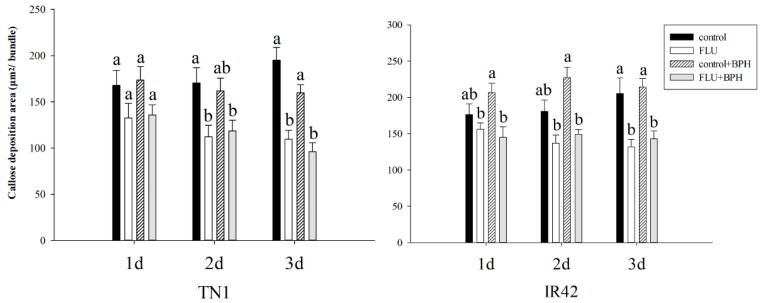
Changes of callose deposition area of TN1 and IR42 rice cultivars after 15 μmol/L FLU treatment and BPH feeding. Different letters in the bars showed the means differed significantly under the same rice cultivar and the same BPH feeding days at the *p* < 0.05 level.

The yellowish green fluorescence light spot showed the callose deposition, which is mainly around the catheter, in the epidermis, mechanical tissue and the xylem tissue (Figure 4). After FLU treatments, callose deposition in rice leaf sheaths was showed in the Figure 5 and after FLU treatments and BPH feeding, the callose deposition area decreased significantly compared to the control (Figure 5). In the TN1 rice cultivar, there was no significant difference between the different treatment groups after 1 day (Figure 6). After 2 days, the callose deposition areas in rice after FLU and FLU + BPH treatments were significantly reduced compared with the control, the rates of decrease were 34.2% and 30.5% respectively (*F* = 4.72, *df* = 3, 39, *p* < 0.05). After 3 days, the callose deposition areas in rice after FLU and FLU + BPH treatments were also significantly reduced compared with the control and the rates of decrease were 43.9% and 50.8% (*F* = 18.75, *df* = 3, 39, *p* < 0.05). In the IR42 rice cultivar, there was no significant difference between the treatment groups and the control after 1 day and 2 days. After 3 days, the callose deposition areas had both significantly reduced compared with the control after FLU and FLU + BPH treatments (*F* = 8.75, *df* = 3, 39, *p* < 0.05). The rates of decrease were 35.8% and 30.4%, respectively. These indicated that FLU inhibited callose deposition in the two rice cultivars and BPH feeding had a different influence on the rice cultivars (Figure 6).

## 4. Discussion

EPG technology allowed the determination of the location of resistance factors and the comparison of the prying and feeding behavior of insecticide-susceptible and resistant insects and was often used as a rapid bioassay for screening resistant plants [29,30]. The feeding behavior of the leafhopper *Scaphoideus titanus* (Hemiptera: Cicadomorpha: Cicadellidae) was analyzed by EPG and the biological significance of the feeding waveform was defined [31]. Backus et al. [30] used a Bennett AC–DC monitor to record the EPG waves of the southern chinch bug, *Blissus insularis* Barber and the western chinch bug, *Blissus occiduus* Barber, and the waveform library was established. Liu et al. found that ABA facilitated rice resistance to BPH [5] and that the biological significance of N4 wave was an index of insect sucking phloem sap, an important marker for measuring plant resistance [32]. Further studies using EPG technology to measure the duration of the N4 wave revealed that ABA treatments reduced the duration of phloem sap ingestion from rice plants [15]. FLU is an ABA inhibitor and we hoped to observe the feeding behavior of BPH under the same conditions in order to further explore the role of ABA in rice resistance to BPH, so we used almost the same concentrations (15, 30 and 60 μmol/L) of FLU. Our results showed that the duration of the N4 wave increased in TN1 rice cultivar after 15 μmol/L FLU treatment but the duration of the N4 wave decreased at higher concentrations of FLU (30 and 60 μmol/L). This indicated that 15 μmol/L FLU facilitated the duration of phloem sap ingestion and rice resistance decreased significantly. In this paper, FLU inhibited the deposition of callose in rice leaf sheaths and led to a reduction of the physical barrier to BPH feeding thereby reducing rice resistance to BPH. However, at higher FLU concentrations (30 and 60 μmol/L), the duration of the N4 wave for the TN1 rice cultivar was shorter and the duration of the N2 wave significantly increased in the TN1 rice cultivar treated with 30 and 60 μmol/L of FLU. This indicated that the taste sensing system of BPH may signal the presence of nutritive compounds that promote feeding as well as toxic compounds. In other words, after rice plants was treated with 30 and 60 μmol/L of FLU, it was not suitable for BPH feeding. The standard rate of different herbicides was different and it was also influenced by the rice cultivars. For example, the liberty aqueous solution with a volume fraction over 0.3% could kill the rice seedlings of non-resistant cultivars but resistant cultivars treated with 0.5% liberty solution at 3–4 leaf stage had a higher yield per plant [33]. As a herbicide, the standard rate of FLU was rarely studied. Xu et al. [34] reported that low concentrations of FLU (1 μmol/L) relieved the inhibition of ABA on maize (*Zea mays* L.) seed germination and promoted the germination of seeds and radicles. In contrast, high concentrations of FLU (100 μmol/L) inhibited maize seed germination and radicle elongation and the albinism of maize leaves occurred after FLU (100 μmol/L) treatment. Some studies had shown that the application of herbicides led to a decline in rice photosynthesis [35,36] and the content of carbohydrates in rice was also reduced [37]. For example, FLU reduced the chlorophyll content, leading to white plant leaves and a decline in photosynthesis and synthesis of organic matter [35]. In this experiment, TN1 rice cultivar treated with 30 and 60 μmol/L of FLU showed a more obvious albino phenomenon (we did not show the image in the paper) but it was not observed in the IR42 rice cultivar. This indicated that FLU with a concentration of over 30 μmol/L could cause phytotoxicity to TN1, while FLU with a concentration of between 15 to 60 μmol/L had no significant effect on the IR42 rice cultivar. Ovarian development was important for the reproduction of BPH. Normally, ovarian development was positively correlated with the fecundity of BPH [24]. In this study, no significant change was found in BPH eggs, oviposition period and ovaries (these indices are closely related to the reproduction of BPH). The results showed that FLU had no effect on the fecundity of BPH. By observing and analyzing the area of callose deposition in TN1 and IR42 rice cultivars, we found that FLU inhibits the deposition of callose. After 15 μmol/L FLU treatments and BPH feeding for 3 days, the areas of callose deposition in the TN1 and IR42 rice cultivars were significantly decreased compared with the control. These indicated that the physical barriers to BPH feeding were significantly reduced after FLU treatment, which was conducive to BPH feeding and reduced rice resistance to BPH.

Many studies have shown that ABA was a negative regulator and involved in plant resistance to disease, for example, Yazawa et al. [38] showed that the number of lesions was reduced before the inoculation of *Magnaporthe grisea* because of the expression of *ABA8ox1* or *OsABI* (G-to-A), which reduced the ABA level or inhibited ABA signaling. Furthermore, ABA promoted rice blast fungus infection in the early stage of infection and enhanced rice sensitivity to the pathogen *Xanthomonas* [39]. In addition, ABA inhibited the formation of callose in *Arabidopsis thaliana* and reduced resistance to disease after bacterial infection [40]. However, ABA also had a positive effect on plants. ABA enhanced plant resistance to salt, drought and cold conditions and played a regulatory role under biological stress [1,13,41,42,43]. For example, in plant-pathogen interactions, ABA enhanced plant resistance to pathogenic bacteria [44] and the mechanisms included the induction of callose deposition [23] and the corpus callosum formed by thickened callose in the cell wall [44]. ABA mediated stomatal closure to prevent the invasion of pathogens [45,46]. Alazem et al. [47] demonstrated that ABA induced resistance to infection with bamboo mosaic virus. The role of ABA in plant resistance to pests is little studied. Liu et al. [15] reported that exogenous ABA increased the callose deposition area in rice, which inhibited BPH feeding behavior and enhanced rice resistance to BPH. The present study showed that FLU, the inhibitor of ABA, facilitated BPH feeding behavior which further proved that ABA enhances rice resistance to pests from another perspective. Our results indicated that FLU had the opposite effects on plant resistance compared to ABA.

Hormonal interaction regulates plant defense responses to abiotic and biotic stresses. The application of ABA inhibited the level of jasmonic acid (JA) in plants, while ABA-biosynthesis inhibition led to an increase in JA levels, confirming an antagonism between ABA and JA in rice roots [48]. In Arabidopsis, the synergistic interaction between ABA and JA could occur through MYC2 (a transcription factor, TF) and its homologs MYC3 and MYC4. The over-expression of MYC2 in Arabidopsis caused sensitivity to ABA and exogenous ABA enhanced the expression of MYC2 in Arabidopsis [49]. It was also a key regulator of plant resistance to pests and activated JA-mediated defense responses [50]. The interaction between ABA and JA signaling pathways could be significant for optimizing plant responses to the combined stress of herbivorous insects and drought, making drought-stressed plants more resistant to insects than well-watered and flooded plants [51]. Salicylic acid (SA) induced plant defense responses to piercing-sucking pests [52] and promoted the production of protein inhibitors, nicotine, agglutinin and other compounds [53,54] and endogenous SA and H_2_O_2_ took part in plant responses to herbivore infestation [55]. Moeder et al. [56] found that SA and ABA had antagonistic effects in the lesion mimic mutant *cpr22* and *ssi4*, when these mutants were transferred from high humidity to low humidity conditions. Both SA and ABA signals were up-regulated and SA signals prevented the downstream ABA signal. In brief, hormonal signaling interactions help plants to defend against a specific attack. As an inhibitor of ABA, FLU interplays with JA or SA or JA and SA. Our experiment demonstrated that callose deposition was inhibited by FLU treatment and that a low concentration of FLU (15 μmol/L, on TN1) promoted BPH feeding behavior but there were no significant influences on BPH fecundity. The results finding that callose deposition in rice cell walls was inhibited by FLU could further confirm that ABA could increase plant resistance to pests and this would help to bring forward a new method for managing BPH and other piercing-sucking pests.

## 5. Conclusions

In conclusion, FLU treatment reduced the area of callose deposition in rice cultivars (TN1 and IR42) and this result was contrary to that of ABA. In terms of feeding behavior, low concentration FLU treatment (15 μmol/L) increased BPH feeding time of sap ingestion in the phloem on TN1 but high concentration FLU treatment (30, 60 μmol/L) achieved the opposite effect. For IR42, there was no significant difference in the duration of sap ingestion. These may be due to the differences between susceptible and resistant cultivars. In addition, our study found that FLU treatment had no significant effect on BPH reproduction. These results further confirmed that ABA enhanced rice resistance to BPH.
